# Izmir Mental Health Cohort for Gene-Environment Interaction in Psychosis (TürkSch): Assessment of the Extended and Transdiagnostic Psychosis Phenotype and Analysis of Attrition in a 6-Year Follow-Up of a Community-Based Sample

**DOI:** 10.3389/fpsyt.2019.00554

**Published:** 2019-08-07

**Authors:** Umut Kırlı, Tolga Binbay, Hayriye Elbi, Marjan Drukker, Bülent Kayahan, Ferda Özkınay, Hüseyin Onay, Köksal Alptekin, Jim van Os

**Affiliations:** ^1^Department of Psychiatry, Faculty of Medicine, Yuzuncu Yil University, Van, Turkey; ^2^Maastricht University Medical Centre, School of Mental Health and Neuroscience, Department of Psychiatry and Psychology, South Limburg Mental Health Research and Teaching Network, Maastricht, Netherlands; ^3^Department of Psychiatry, Faculty of Medicine, Dokuz Eylul University, Izmir, Turkey; ^4^Department of Psychiatry, Faculty of Medicine, Ege University, Izmir, Turkey; ^5^Department of Medical Genetics, Faculty of Medicine, Ege University, Izmir, Turkey; ^6^Department of Psychosis Studies, Institute of Psychiatry, King’s College, King’s Health Partners, London, United Kingdom; ^7^Department of Psychiatry, Brain Centre Rudolf Magnus, Utrecht University Medical Centre, Utrecht, Netherlands

**Keywords:** extended psychosis phenotype, transdiagnostic psychosis phenotype, epidemiology, representative community-based sample, neighborhood-level measures, gene-environment interactions

## Abstract

**Objective:** TürkSch is a prospective, longitudinal study in a representative community sample (İzmir, Turkey), consisting of several data collection stages, to screen and follow-up mental health outcomes, with a special focus on the extended and transdiagnostic psychosis phenotype. The aim of the present paper is to describe the research methodology, data collection results, and associations with noncontact and refusal in the longitudinal arm.

**Methods:** Households were contacted in a multistage clustered probability sampling frame, covering 11 districts and 302 neighborhoods at baseline (n = 4,011) and at 6-year follow-up (n = 2,185). Both at baseline and at follow-up, participants were interviewed with the Composite International Diagnostic Interview. Participants with probable psychotic disorder were reinterviewed with the Structured Clinical Interview for Diagnostic and Statistical Manual of Mental Disorders, Fourth Edition (SCID)-I either at the hospital or at the participant’s residence. Relevant neighborhood-level measures were assessed in a separate sample (n = 5,124) in addition to individual-level measures. Candidate gene-by-environment interactions were investigated using two nested case-control studies.

**Results:** Individuals with a mental health problem had lower refusal rates. Older and lower educated individuals had a lower probability of noncontact.

**Discussion:** The TürkSch study has an advanced design to meet the challenges of evaluating the multidimensional etiological and phenomenological nature of the extended and transdiagnostic psychosis phenotype.

## Introduction

After nearly two decades of epidemiological studies, there is evidence suggesting that psychosis is distributed as a spectrum (1). The spectrum of psychosis extends from the clinical psychotic syndrome to nonpsychotic diagnoses with a degree of psychosis admixture and, finally, to nonclinical populations with subthreshold psychotic experiences (2–4). Thus, the *extended psychosis phenotype* is the range from psychotic experiences (PEs) gradually blending into psychotic disorders (PDs) (2).

The majority of individuals with PEs have a diagnosis of nonpsychotic disorder. Conversely, a high prevalence of PEs has been demonstrated in individuals with nonpsychotic disorders where they can be considered markers of clinical severity (5). Furthermore, PEs and nonpsychotic disorders have been shown to predict subsequent occurrences of each other, bidirectionally (6). It has been suggested that these findings point to a *transdiagnostic psychosis phenotype* in the general population (7).

To date, the definition of PEs and the type of screening instrument used have varied across studies (8), contributing to heterogeneity of results in this area. In the majority of studies, definitions of PEs were based on attenuated forms of delusional thinking and hallucinatory perceptions (1, 9, 10). However, negative, disorganization, and affective dimensions of psychosis have been identified in addition to the positive dimension. These dimensions should also be taken into account (7).

Longitudinal studies have demonstrated that PEs are mostly transitory. Persistent PEs have been associated with a greater risk of need for care (11) and prolonged exposure to environmental risks (childhood adversity, minority position, discrimination, urban upbringing and residency, stress in a wider social environment, substance misuse, etc.), possibly interacting with a genetic liability (12). Hence, a growing number of studies have tried to disentangle the components of interactions between genes and the environment underlying the *extended and transdiagnostic psychosis phenotypes* (11, 13–15). Although these studies provided new insights, further studies are required to strengthen the current evidence and generate novel findings. To date, several genes have been associated with the pathophysiology of psychosis, including neuregulin 1(NRG1), brain-derived neurotrophic factor (BDNF), and catechol-O-methyltransferase (COMT), along with the others (16–18). Furthermore, studies have shown some effect of these genes on functional brain abnormalities that may be associated with psychosis susceptibility (19–23). However, evidence on the association between these genes and schizophrenia was inconsistent (24–27), and no polymorphisms of these genes have reached genomewide significance in schizophrenia (28, 29). Nevertheless, variants of the BDNF and the NRG1 have been associated with dimensions of psychosis across diagnostic boundaries (30–33). Furthermore, gene-environment interactions involving these genes and different levels of the extended psychosis phenotype have been documented (34–36). Although the COMT was associated with a small effect on stress reactivity, no main effect was found at the level of the extended psychosis phenotype (37). Given these reports, there is a need to extend the research on gene-environment interactions to the extended and transdiagnostic psychosis phenotype.

There is strong evidence that variables that include phenomenological expression, neurocognitive functioning, socioenvironmental and genetic liabilities are shared among PEs and PDs across traditional diagnostic boundaries (7). Therefore, an approach based on searching for differences between individuals with a distinct disorder category (e.g., schizophrenia) and the healthy population (which may also include individuals with PEs) may mask some of the relevant associations with the psychosis spectrum (38). From this point of view, it is important to take the full spectrum of psychosis (including both clinical and subclinical symptoms in non-help-seeking individuals) into account.

In cross-sectional studies, a part of the associations may be missed because symptom presence and severity of psychosis are subject to fluctuations over time (39). Longitudinal studies assessing the psychosis phenotype along a severity spectrum can shed light on changes over time. This framework is also useful to detect the factors predisposing to poor outcome in the psychosis spectrum (40).There is preliminary evidence that different types of risk factors may be leading to a differential expression of symptom dimensions (41). Therefore, a multidimensional assessment of the psychosis spectrum is needed to identify nonshared factors associated with specific psychosis dimensions and shared factors underlying the transdiagnostic psychosis phenotype (7). This approach also brings the opportunity to further investigate preliminary findings suggesting an impact of environmental and genetic load on the connectivity between different dimensions of psychosis (42).

Previous studies in this area have usually defined the clinical end of the extended psychosis phenotype based on self-report or lay-interviewer assessments. Relatedly, the clinical outcomes mostly have been conceptualized as a shift on the dimensional scale of frequency/duration of an attenuated positive symptom, representing a quantitative rather than a qualitative measure. At the same time, the unitary representations of the poor outcome of the spectrum have probably caused an obstacle to identify possible associations of the risk factors with different types of outcomes (43). Therefore, clinical reinterviews with individuals with a positive screening of psychosis are required to define more valid and efficient clinical outcomes from a multidimensional psychopathological point of view. Furthermore, most studies were not designed to specifically study the psychosis spectrum phenotype. Thus, risk factors included were not selected for their association with psychosis. In addition, factors in the wider social environment, such as neighborhood-level risk factors, were not included, and studies were not genetically sensitive, with a few notable exceptions (44–46). There is robust evidence that genetic and socioenvironmental factors, both at the individual and the neighborhood levels, interact with each other in the development and course of the psychosis spectrum (47, 48). To study gene-environment interactions when analyzing the development and course of the extended and transdiagnostic psychosis phenotype, simultaneous assessments of the genetic and the environmental factors both at the individual level and at the level of the wider social environment are required. The Izmir Mental Health Survey for Gene-Environment Interaction in Psychosis (TürkSch) was therefore conducted to provide new insights into and knowledge of the *extended and transdiagnostic psychosis phenotype*, and to identify social-environmental risks in interaction with genetic background (47, 49, 50).

The present paper describes the methods of the TürkSch follow-up. Furthermore, dynamic transitions over time in the extended psychosis phenotype are presented. Finally, the associations between various variables and noncontact/refusal in the longitudinal arm are analyzed.

## Methods

### Overview of the Design of the TürkSch Cohort

TürkSch is a prospective, longitudinal study to screen and follow-up mental health outcomes in a representative general population sample of Izmir, Turkey. The TürkSch consists of two separate assessments (T_1_ and T_2_) and several stages of data collection. The study assessed the prevalence of the extended psychosis phenotype. In addition, associations between various individual-level variables and the extended psychosis phenotype were investigated (*stage 1, T* *_1_*). Associations between neighborhood-level variables (e.g., socioeconomic deprivation and social capital of neighborhoods) and the extended psychosis phenotype were assessed by a separate data collection independent from the main data collection (*stage 2, T* *_1_*, *n = 5,124*). Furthermore, a nested case-control study recruited individuals with PEs and PDs and individuals with no psychotic symptoms from stage 1 and included blood sampling for analysis of gene-environment interactions (*stage 3, T* *_1_*).

Six years after baseline, mental health and environmental exposure were assessed (*stage 4, T* *_2_*). Finally, a longitudinal nested case-control study recruited individuals using the results of *stages 1 and 4*, and blood samples were collected for further genetic analysis (*stage 5, T* *_2_*). The TürkSch study was approved by the institutional ethics review board of Ege University, Turkey, and is compliant with the precepts of the Declaration of Helsinki. Each participant provided written informed consent for the examination and procedures.

### Sample

At baseline, the Turkish Institute of Statistics (TurkStat) randomly selected 6,000 households representative of the Izmir metropolitan area using a multistage sampling procedure stratified by urbanicity in four categories and covering 11 districts and 302 neighborhoods. Addresses were contacted in person. One household member aged between 15 and 64 years and available to complete the interview was randomly selected using the Kish within-household sampling method (51). Out of 6,000 addresses, 5,242 households were eligible for interview. A total of 4,011 individuals were successfully interviewed, yielding a response rate of 76.5% in *stage 1*. Response was higher in older age groups and in females. Full details on the Izmir metropolitan area, sampling, representativeness, instruments, procedures of T_1_, and the map of neighborhoods included can be found in a previous article (50). Participants and addresses of T_1_ formed the targeted population for T_2_.

### Fieldwork

Follow-up assessments (T_2_) were performed approximately 6 years after the baseline assessments (T_1_). To optimize response, T_2_ fieldwork was spread over a relatively long period (2 years) so that there was sufficient time to recontact potential respondents. At T_2_, addresses of T_1_ participants were visited in person by trained lay interviewers with a brochure reminding the study, providing results from baseline, seeking participation for a new interview, and explaining the study goals in detail. The brochure also referred to a website, full names of the study team, and a phone number of the research office. If the participant could not be reached at the address, the study team telephoned the participants using numbers from T_1_. In these calls, the team ascertained whether the participant was reachable, and if this was the case, appointments were made for face-to-face interviews. Any contact information of the participants who could not be reached was collected by asking neighbors in the area or the neighborhood authorities. If additional information was obtained, the person was contacted at the new contact address. Any T_1_ participant was defined as unreachable at T_2_ after at least three consecutive visits to the address.

### Interviewers, Interviewer Training, and Quality Control

At T_1_, lay interviewers had at least high school education, a health-related profession, and/or were experienced in doing field surveys. At T_2_, lay interviewers were psychology graduates. At T_2_, both the lay interviewers and the psychiatrist who conducted the clinical reinterviews (UK) had not participated in T_1_ and thus were blind to baseline results. At both assessments, lay interviewers had a 2-week formal training that included basic information on common mental disorders, symptom dimensions of psychosis, ethical aspects of the project, and practical training. The fieldwork was closely monitored by the study team (UK, TB, HE, BK, KA). Each interview at T_1_ and T_2_ was conducted according to a standard procedure, with recording and quality coding. If any of the three following problems were determined: i) the quality of the interview was considered low; ii) any missing value was present; and iii) there was a doubt whether the endorsed symptom was a true symptom, as described later, a phone call or a second visit (T_1_, n = 392; T_2_, n = 560) was planned by the study team. The missing values still present after the second visit were assessed by the psychiatrist after the clinical reinterview.

### Screening Instrument

To assess mental health outcomes, screening was based on the relevant sections of the Composite International Diagnostic Interview (CIDI) 2.1 (52). The CIDI is a fully structured interview developed by the World Health Organization (WHO) (53) and has been used in various surveys around the world, including ones in Turkey (54–56). Primarily designed for use in epidemiological studies of mental disorders, the CIDI can be used by both clinicians and trained interviewers. CIDI-based screening of symptoms provides diagnoses in accordance with the definitions and criteria of the International Classification of Diseases, 10th Revision (ICD-10), and the *Diagnostic and Statistical Manual of Mental Disorders, Fourth Edition* (DSM-IV), along with information about frequency, duration, help seeking, severity of symptoms, and psychosocial impairment. CIDI 2.1 has organic exclusion rules, which are used to construct final diagnoses, for each endorsed symptom, to ascertain that symptoms were not exclusively caused by a somatic cause, an injury, or use of drugs, alcohol, or medication. Previous research reported acceptable-to-good concordance between the CIDI 2.1 diagnoses and blind clinical diagnoses (57–59). The CIDI was found to have excellent interrater reliability in almost all sections, with κ values ranging from 0.67 to 0.97 (60). In particular, κ for agreement between clinicians for delusions and hallucinations was found to be 0.85 and 0.87, respectively. Furthermore, the sensitivity of the CIDI was found to be higher than its specificity for both delusions (0.93 vs. 0.55) and hallucinations (0.86 vs. 0.50) (61). The reliability and validity functions of the Turkish version of the CIDI were studied as part of an international study (62).

Mental health screening at both T_1_ and T_2_ included CIDI screening sections on alcohol and substance-related disorders, depressive and dysthymic disorders, manic and bipolar affective disorders, schizophrenia and other PDs, posttraumatic stress disorder, and two final sections containing concluding questions, interviewer observations, and interviewer ratings (50). The time frame of the T_2_ CIDI interview was the last 6 years.

### Assessment of the Dimensions of Psychosis

*Assessment of the positive dimension* was based on 14 CIDI delusions items (G1, G2, G3, G4, G5, G7, G8, G9, G10, G11, G12, G13, G13b, and G14) and five CIDI hallucinations items (G17, G18, G20, G20C, and G21). All items were rated dichotomously, indicating presence or absence. Rating of the PEs can be difficult because sometimes individuals can be describing a plausible event or a religious or superstitious belief that in the CIDI may be rated as a PE. Therefore, the following procedure was followed. First, during the interview, each time a participant endorsed a CIDI PE, the participant was asked to give an example, which was written down verbatim by the interviewer for later review with the mental health clinician on the team. All CIDI interviews were reviewed by the study team. When it was not clear whether the participant had truly endorsed a positive PE, the participant was recontacted by a clinician over the telephone to confirm the PE. Thus, delusional and hallucinatory experiences were coded positive if the team clinician confirmed the PE at review. Our results showed that the interrater reliability of the CIDI psychosis section had a κ value of 0.45 at T_1_ (50) and 0.67 at T_2_.

*Assessment of the negative and disorganization dimensions* was based on the CIDI P section, which is on interviewer observations. The negative dimension was based on four symptom items (flat affect, slow speech, poverty of speech, and impaired ability to initiate activity), and the disorganization dimension was based on three symptom items (neologism, thought disorder, and hallucinatory behavior).

*Assessment of the affective dimension* was based on CIDI section E (depressive and dysthymic disorders) and section F (manic and bipolar affective disorders). For depression, participants were asked if they had experienced an episode lasting at least 2 weeks during which they felt depressed or had a lack of interest. If endorsed, participants were asked if, during this period, they had experienced lack of energy, appetite change, sleep problems, being slow or restless, feelings of worthlessness or guilt, decreased self-esteem, trouble thinking or indecisiveness, and thoughts of death. For manic and hypomanic episodes, participants were asked whether they had experienced elevated mood or irritability for a period of at least consecutive days either noticed by others or causing problems. If this was the case, participants were asked if, during this period, they had experienced excessive goal-directed activity, psychomotor agitation, spending sprees, sexual indiscretions, increased talkativeness, flight of ideas, loss of normal social inhibitions, increased self-esteem or grandiosity, decreased need for sleep, and distractibility. For both depressive and manic episodes, the final assessment included questions on probable association of symptoms with substance use or physical illness, help seeking due to symptoms, the route of help seeking, clinician diagnosis, and treatment history. All responses were reevaluated by a team of clinicians. Depressive episode and hypomanic/manic episode were coded positive in accordance with the definitions and criteria of DSM-IV.

### Diagnostic Interviews and Construction of the Extended Psychosis Phenotype

At both T_1_ and T_2_, sections were devoted to define patterns of help seeking for mental health problems. Questions included any self-report mental problem, help seeking for a mental problem, the route of help seeking, the probable outcome of the help seeking (diagnosis), and prescribed medicines and any hospitalization during the time frame (lifetime and last 12 months at T_1_ and last 6 years at T_2_). If this was the case, the person was asked for permission to contact the clinician involved in the diagnosis or the treatment of the participant to verify the diagnosis and review case material.

A measure of impairment associated with PEs was defined using CIDI items G25 (duration of the PE: between 1 day and 6 months or more), G26, G28, G29, and G29A (level of dysfunction) and G16 and G23 (told doctor about psychotic beliefs) (47, 49). Furthermore, *a probable PD case* was defined if any of the following screening findings were endorsed:

1. Any self-reported diagnosis of psychotic or bipolar disorder.
2. Any self-reported hospitalization due to a mental health problem.
3. Any self-reported medication of any antipsychotic (typical or atypical) and/or lithium or mood-stabilizing anticonvulsant drugs.
4. In the CIDI section F for bipolar disorder: a lifetime manic episode.
5. In the CIDI section G for positive dimension: any clinically relevant positive PE (led to dysfunction or help seeking) or at least three symptoms regardless of clinical relevance. If the participant had a clinically relevant positive PE at T_1_, he/she was directly defined as a *probable case* regardless of the CIDI endorsement at T_2_.
6. In the CIDI section P for negative and disorganization dimensions: a rating of positive formal thought disorder, negative symptoms, behavior that suggests that the person is having hallucinations, or catatonic symptoms, or the interviewer comments were indicative of a PD.

If a participant was deemed to have a diagnosis of *probable PD* according to the algorithm mentioned, the participant was recontacted by the team psychiatrist and invited to the hospital for a clinical evaluation with the Structured Clinical Interview for DSM-IV (SCID) (63). When the participant did not attend the hospital, clinical interviews were conducted at the participant’s residence by the psychiatrist. Thus, 225 participants at T_1_ and 263 participants at T_2_ were clinically reinterviewed to identify participants with PD.

**Figure 1:**
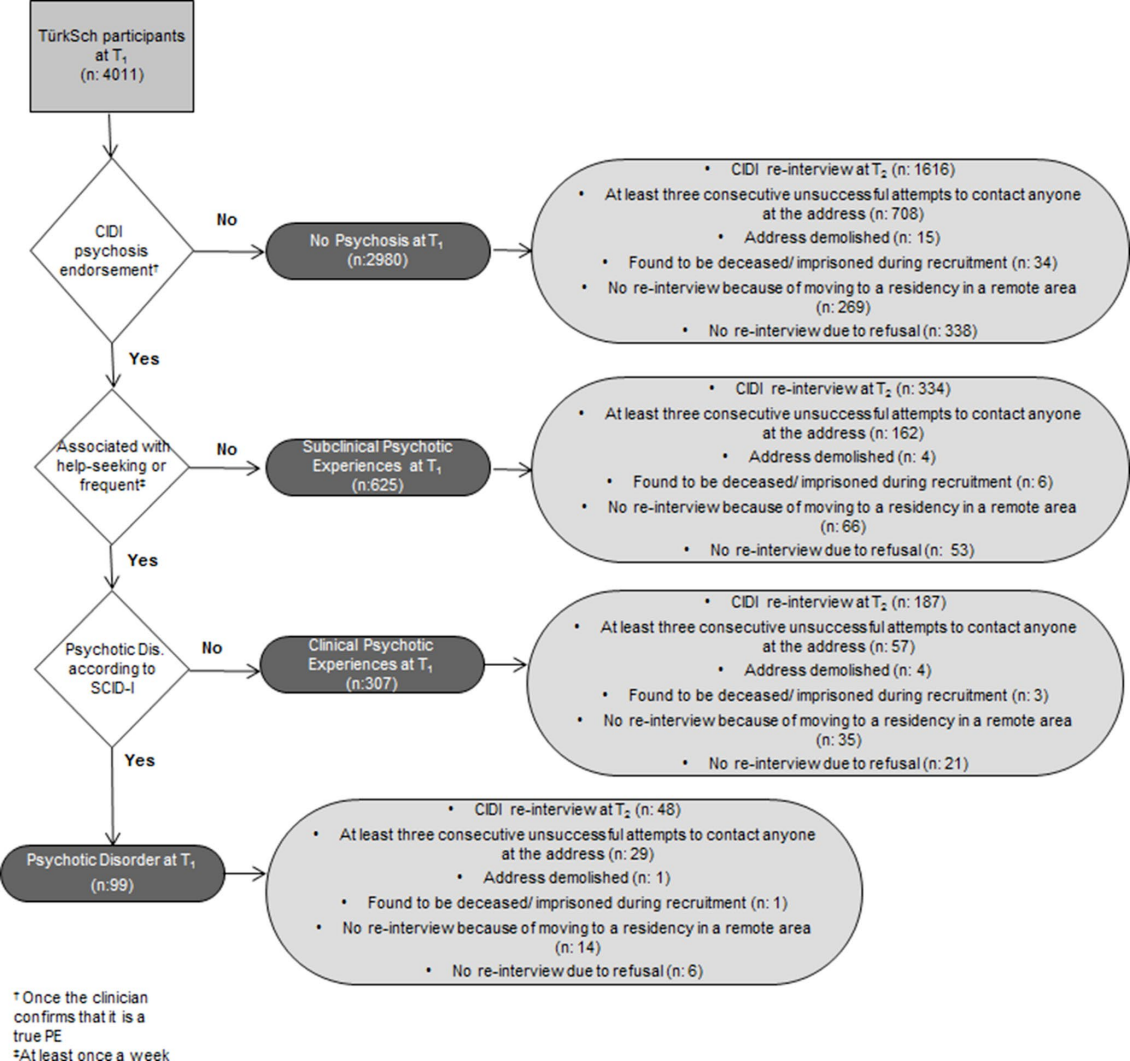
Assessment of the extended psychosis phenotype and data collection results at follow-up.

An* extended psychosis phenotype* variable was constructed including four categories using the SCID results and *the measure of impairment associated with PEs*. *The psychotic disorder group* included all individuals diagnosed with any DSM-IV disorder with psychotic features. *The* *clinical PE group* included individuals who had a CIDI PE leading to any of the seven CIDI impairment items but who did not have a diagnosis of a PD. *The subclinical PE group* included individuals with a CIDI PE not leading to any distress, impairment, or help seeking. All other individuals were included in the *no psychosis* category. The flowchart of the assessment of the extended psychosis phenotype and the numbers of individuals in each group are presented in **Figure 1**.

### Assessment of Environmental Exposures at the Individual Level

A sociodemographic questionnaire was included at T_2_ to determine temporal changes in background characteristics (age, educational status, marital status, employment, socioeconomic status, health insurance, housing, and monthly household income). The T_1_ interview also included educational and occupational status of parents, birth year of parents, migration pattern and probable reasons for migration, ethnic group, and any history of early childhood adversity (e.g., parental loss, divorce, separation). Socioeconomic status was estimated using profession and recoded into four ordinal categories (1: I and II professionals and IIIA nonmanual high employees, 2: IIIB nonmanual low employees and V and VI skilled workers and technicians, 3: IVA, IVB, and IVC owners of small businesses, and 4: VIIA and B manual workers (49, 64).

The variable *traumatic events* was obtained using the posttraumatic stress disorder section of the CIDI. The events were war experience, life-threatening accident, fire, flood or other natural disaster, witnessing someone being badly injured or killed, rape, sexual molestation, and being physically attacked or assaulted. Furthermore, the interview included the *List of Threatening Life Events* (65) so as to cover most of the stressful life events experienced by individuals. Threatening life events included a serious illness, injury, or an assault (suffering or happening to a close relative); death of a relative or a close friend, divorce, separation, serious problems with a relative/neighbor/close friend; being dismissed from a job, unemployment, major financial problems; and police/court appearance. Time frame was the last 6 years.

Alcohol, *Cannabis*, and other substance uses were assessed using screening questions on CIDI alcohol and substance-related disorders section (66). Using information from both T_1_ and T_2_, the continuum of alcohol, *Cannabis*, and other substance uses during the follow-up period was defined.

### Assessment of Neighborhood-Level Measures

At T_1_, urbanicity (birth place, places of residence at age 0–15 years, and current place of residence) was assessed. In a separate sample, socioeconomic deprivation and the social capital of the resided neighborhoods were assessed (50). T_2_ assessment included questions on changes in place of residence. Furthermore, the description of the visited neighborhood and building was coded by the interviewer in five categories (village/slum/semi-urban/urban/luxury area). *Urbanicity* of the place of residence was defined using the classification of the Turkish Institute of Statistics (TurkStat). The classification depended on the level of organized features of streets and buildings (regularity of sidewalks, status of road, completeness of drainage system, and quality of outer paintings of buildings, etc.) (47). *Social capital of the neighborhood* was assessed using two assessments: informal social control and social disorganization. Questions on informal social control were derived from the Sampson collective efficacy scale (67), adapted for use in the Turkish population (47). The informal social control scale measures the willingness to intervene in hypothetical neighborhood threatening situations, for example, in the case of children misbehaving. The items were assessed using a 5-point Likert scale ranging from *strongly agree* to *strongly disagree*. Eight items assessing social disorganization were derived from the McCulloch instrument (68, 69). Respondents rated the frequency of certain scenarios occurring in their neighborhood (presence of graffiti, teenagers on street, vandalism, attacks due to race or skin color, other attacks, and burglary and the theft of, or from, vehicles). Each item was assessed using a 4-point Likert scale ranging from *very common* to *not at all common* (47).

### Assessment of Familial Measures

Using questions derived from the Family Interview for Genetic Studies (70), history of mental disorders in the father, mother, siblings, and offspring was assessed. Thus, *a family history of mental disorders variable* was defined and coded guided by previous literature (71): 0 = No or undefined family history of mental disorders; 1 = Common mental disorder (depression/anxiety disorders/obsessive compulsive disorder/posttraumatic stress disorder/substance misuse without a history of hospitalization for the psychiatric condition, or a history of a completed suicide) in at least one family member but no severe mental illness; 2 = Severe mental illness (bipolar disorder/psychotic disorder/hospitalization/completed suicide) in at least one family member (49).

### Blood Sampling and Assessment of Candidate Gene-Environment Interactions

At T_1_, a nested case-control study (*stage 3*, n = 366) recruited individuals with PEs and PDs as well as individuals with no psychotic symptoms to investigate gene-environment interactions in the extended and transdiagnostic psychosis phenotypes. In this subgroup, *COMT* val158met (rs4680) and *BDNF* val66met (rs6265) polymorphisms were assessed besides the clinical reappraisals and exposures previously mentioned.

At T_2_, environmental exposures for the last 6 years were assessed, followed by clinical reappraisals in eligible individuals (n = 254).

At T_2_, a subsample of subjects was selected for a second nested case-control study (*stage 5*) using the results of both T_1_ and T_2_. First, 200 individuals with any psychotic symptoms (either PE or PD) at either T_1_ or T_2_ were randomly selected. Then, these individuals were matched with 200 individuals who participated in both T_1_ and T_2_ and had no psychotic symptoms (neither PE nor PD) during the follow-up period. Matching variables were age, gender, and neighborhood. The selected individuals were asked to provide a blood sample for further genetic analysis and clinical reappraisals. A total of 174 individuals with any psychotic symptom (61 with PD; 113 with PE) and 151 individuals with no psychotic symptoms during follow-up provided a blood sample. In light of the previous results, previously mentioned, we decided to evaluate the *BDNF* and the *NRG1* as candidate genes rather than the *COMT* gene in the whole gene sequence analysis procedure in T_2_. Results were evaluated considering the environmental exposure results at both T_1_ and T_2_.

### Statistical Analysis

To evaluate differential attrition over time, a two-step analysis was performed. First, a multinomial logistic regression model was performed (dependent variable with three categories: 0 = respondent, 1 = noncontact, 2 = refusal) to examine the role of baseline sociodemographics, psychopathology, and environmental exposure variables on the association with the two types of attrition, separately. These associations were expressed as relative risk ratios (RRR) and their 95% confidence intervals. Then, the overall effects of the previously mentioned variables on attrition were tested using chi-square tests and the relevant effect size measure (Cramer’s *V*). Cramer’s *V* equals 0 when there is no relationship between the two variables and has a maximum value of 1. A larger value for Cramer’s *V* indicated a stronger relationship between the variables.

## Results

### Data Collection Results

**Figure 2:**
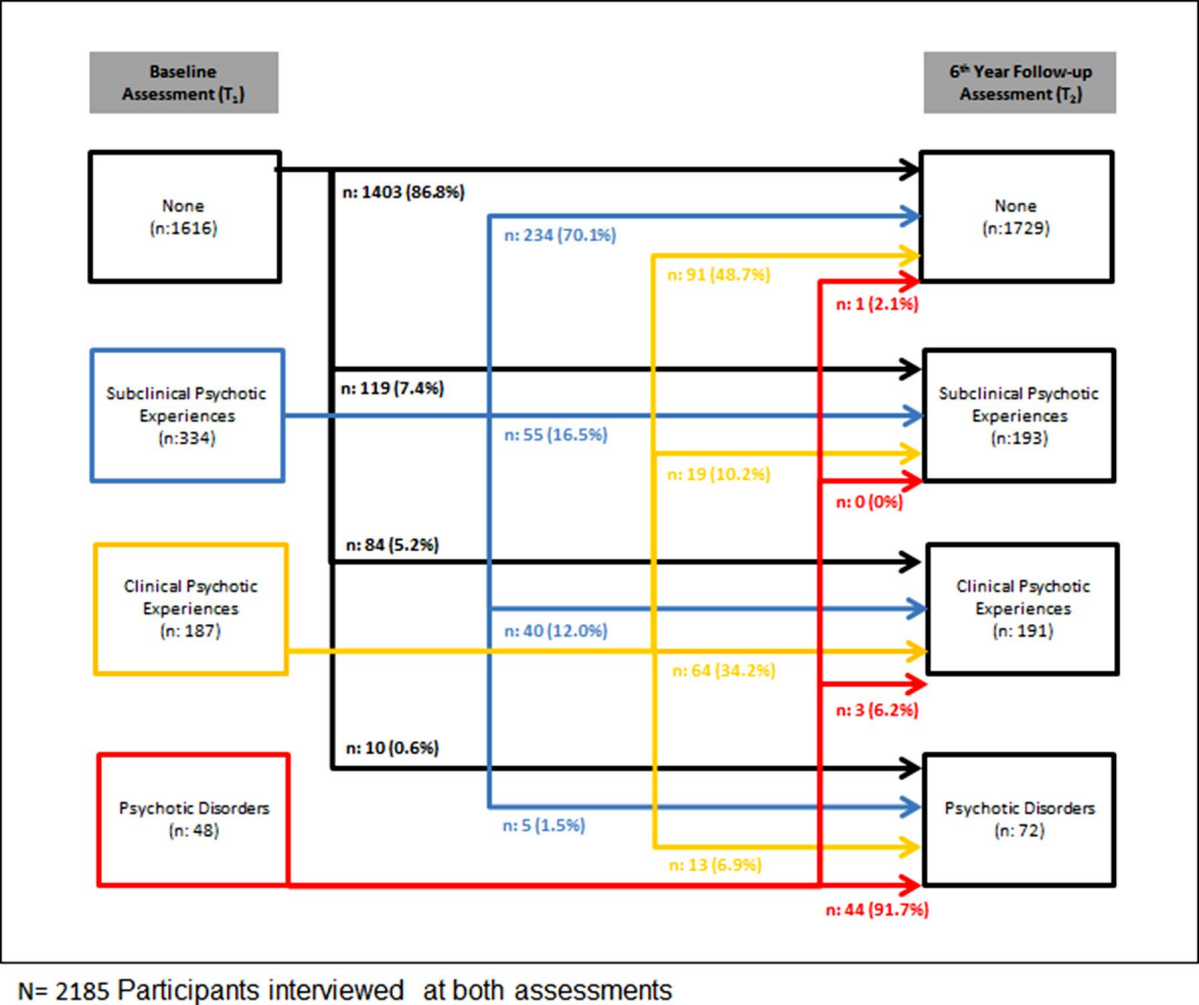
Dynamic transitions over time in the extended psychosis phenotype.

At T_2_, 954 individuals from the baseline sample could not be contacted (i.e., after at least three consecutive attempts to contact anyone at the address), and 386 individuals were lost to follow-up because of moving to a residency in a remote area. Forty-four individuals were deceased or imprisoned, and 24 addresses were demolished. Furthermore, 418 individuals refused to participate in the follow-up assessment. As a result, a total of 2,185 individuals were successfully reinterviewed at T_2_. **Figure 1** shows details of the data collection results at T_2_ stratified by the baseline position across the extended psychosis phenotype. Dynamic transitions over time in the extended psychosis phenotype are presented in **Figure 2**.

### Associations With the Two Types of Attrition (Noncontact and Refusal)

Attrition due to noncontact was significantly higher in individuals who were younger, nonmarried, more highly educated, non-help-seeking, without a valid health insurance, and using *Cannabis* and regular alcohol. The probability of refusal was significantly higher in individuals who were in paid employment, single, more educated, and with higher socioeconomic status. Furthermore, refusal was lower in individuals with a baseline mood disorder, a baseline clinical PE, a history of a traumatic event, and a family history of a severe mental illness (**Table 1**). However, analysis of overall effect on attrition showed that the associations with any independent variable had a Cramer’s *V* value lower than 0.09, indicating very small effect sizes.

**Table 1:** Association between the two types of attrition (refusal/noncontact) and baseline characteristics.

	Respondents	Noncontact		Refusal		Overall effect on attrition	
	n (%)	n (%)	RRR (95%CI)	n (%)	RRR (95%CI)	χ² (琼df)	Cramer’s *V*	
Sociodemographic characteristics							
**Sex**							
Male	890 (52.9)	612 (36.4)	1	181 (10.7)	1	3.0 (2)	0.03
Female	1,295 (55.6)	796 (34.2)	0.89 (0.78–1.02)	237 (10.2)	0.89 (0.72–1.11)		
**Age**							
46–65	699 (49.0)	591 (41.5)	1	135 (9.5)	1	48.9 (4)**	0.08
31–45	750 (54.7)	468 (34.1)	1.31** (1.10–1.56)	153 (11.2)	1.15 (0.89–1.49)		
15–30	736 (60.6)	349 (28.7)	1.78** (1.50–2.10)	130 (10.7)	1.09 (0.84–1.42)		
**Educational level**							
Basic	966 (58.2)	543 (32.7)	1	151 (9.1)	1	17.9 (4)**	0.05
High school	360 (52.0)	261 (37.7)	1.28** (1.06–1.56)	71 (10.3)	1.26 (0.92–1.71)		
University	859 (51.8)	604 (36.4)	1.25** (1.07–1.45)	196 (11.8)	1.45** (1.15–.1.83)		
**Marital status**							
Married	1,638 (57.7)	912 (32.1)	1	289 (10.2)	1	52.0 (4)**	0.08
Single	458 (47.0)	400 (41.0)	1.56** (1.34–1.83)	117 (12.0)	1.44** (1.14–1.83)		
Divorced	89 (45.2)	96 (48.7)	1.93** (1.43–2.61)	12 (6.1)	0.76 (0.41–1.41)		
**Ethnicity**								
Turkish	1,840 (54.6)	1,175 (34.8)	1	358 (10.6)	1	1.2 (2)	0.02
Non-Turkish	345 (54.1)	233 (36.5)	1.05 (0.88–1.26)	60 (9.4)	0.89 (0.66–1.20)		
**Employment status**							
In paid employment	1,020 (54.4)	639 (34.0)	1	218 (11.6)	1	5.9 (2)	0.04
Not in paid employment	1,165 (54.6)	769 (36.0)	1.05 (0.92–1.21)	200 (9.4)	0.80* (0.65–0.99)		
**Health insurance**							
Present	1,949 (55.6)	1,174 (33.5)	1	381 (10.9)	1	32.3 (2)**	0.09
Absent	236 (46.6)	234 (46.1)	1.64** (1.35–2.00)	37 (7.3)	0.80 (0.55–1.15)		
**Socioeconomic status**							
1	466 (54.4)	280 (32.7)	1	111 (12.9)	1	17.6 (6)**	0.05
2	585 (54.1)	382 (35.3)	1.08 (0.89–1.32)	115 (10.6)	0.82 (0.61–1.10)		
3	352 (51.9)	246 (36.3)	1.16 (0.93–1.44)	80 (11.8)	0.95 (0.69–1.10)		
4	782 (56.1)	500 (35.9)	1.06 (0.88–1.28)	112 (8.0)	0.60** (0.45–0.80)		
Baseline clinical characteristics							
**Mental help seeking**							
None	1,872 (53.7)	1,242 (35.7)	1	370 (10.6)	1	5.9 (2)	0.04
Yes	313 (59.4)	166 (31.5)	0.79* (0.65–0.97)	48 (9.1)	0.77 (0.56–1.07)		
**Baseline mood disorder**							
None	1,783 (54.2)	1,143 (34.8)	1	363 (11.0)	1	7.5 (2)*	0.04
Yes	402 (55.7)	265 (36.7)	1.02 (0.86–1.22)	55 (7.6)	0.67** (0.49–0.91)		
**Baseline Cannabis**							
None	2,161 (54.7)	1,377 (34.8)	1	413 (10.5)	1	7.4 (2)*	0.04
>5 times	24 (40.0)	31 (51.7)	2.02** (1.18–3.46)	5 (8.3)	1.09 (0.41–2.87)		
**Baseline alcohol**							
<Once a week	2,055 (55.2)	1,285 (34.5)	1	383 (10.3)	1	11.0 (2)**	0.05
At least once a week	130 (45.1)	123 (42.7)	1.51** (1.17–1.95)	35 (12.2)	1.44 (0.97–2.13)		
**Traumatic event**							
None	1,383 (53.5)	903 (34.9)	1	298 (11.5)	1	9.9 (2)**	0.05
At least one	802 (56.2)	505 (35.4)	0.96 (0.83–1.10)	120 (8.4)	0.69** (0.55–0.87)		
**Family history**							
None or unknown	1,903 (54.0)	1,245 (35.3)	1	379 (10.7)	1	9.4 (4)	0.03
Common mental disorder	222 (56.8)	132 (33.8)	0.90 (0.72–1.14)	37 (9.5)	0.83 (0.58–1.20)		
Severe mental illness	60 (64.5)	31 (33.3)	0.78 (0.50–1.22)	2 (2.2)	0.16* (0.04–0.68)		
**Extended psychosis phenotype**							
No PE	1,616 (54.2)	1,026 (34.4)	1	338 (11.4)	1	19.1 (6)**	0.05
Subclinical PE	334 (53.4)	238 (38.1)	1.12 (0.93–1.34)	53 (8.5)	0.75 (0.55–1.03)		
Clinical PE	187 (60.9)	99 (32.3)	0.83 (0.64–1.07)	21 (6.8)	0.53** (0.33–0.85)		
Psychotic disorder	48 (48.5)	45 (45.4)	1.47 (0.97–2.23)	6 (6.1)	0.59 (0.25–1.40)		

*p<0.05, **p<0.01

Abbreviations: CI, confidence interval; RRR, relative risk ratio

## Discussion

The TürkSch study was conducted in a general population sample, representative of the urban and rural areas of the city of Izmir, representing the third most industrialized area of Turkey. The primary focus of the study was the extended and transdiagnostic psychosis phenotypes, which were prospectively evaluated. Therefore, risk factors were chosen for their association with psychosis. Furthermore, the design of the study enabled us to assess the different symptom dimensions of psychosis (positive/negative/disorganization/affective). The sample size was relatively large and included both help-seeking and non-help-seeking individuals, so we were able to prevent help-seeking bias (43). Furthermore, diagnostic interviews were performed by psychiatrists with individuals with positive screening results. Therefore, we could assess psychotic outcomes along a spectrum, including both clinical and subclinical levels in the same sample. The assessments included family history and environmental exposures both at the individual and the neighborhood levels. The inclusion of candidate gene-based genetic analysis provided the opportunity to longitudinally evaluate specific gene-environment interactions in psychosis along a spectrum of severity.

The results from this study may have important implications. For the last decades, efforts to construct a new nosology of the psychosis spectrum, consisting of dimensional liabilities that cut across current categories, have gained interest (72) mainly because of the low validity of the current diagnostic categories and the associated stigmatization and diminished expectations from the interventions (40). This study yields high-quality data to elucidate the factors underlying specific dimensions of psychosis and the general psychosis factor, encompassing clinical and subthreshold severity levels (73). Thus, this study can contribute to efforts to better conceptualize psychosis. By investigating the complex interactions between different psychosis dimensions, genetic liability, and exposures involving the microlevel and the wider social environment, this study may provide novel information on causation and early intervention strategies (74). Finally, assessment of the factors in the wider social environment can provide a base for community-based interventions in addition to individual-level interventions.

The following limitations of the study should be noted. First, the relatively long period of time between the two data collection points (6 years) might have decreased our ability to establish the course of psychosis in detail (75). Second, as with most longitudinal studies with general population-based samples, the possibility of bias caused by differential attrition over time was a limitation. However, the dropout rate of participants in the current study is similar to that in studies using a similar methodology (11, 15). Furthermore, the comparison of baseline characteristics among respondents, refusals, and noncontacts showed no large differences. Third, the two nested case-control studies (stages 3 and 5) in which gene-environment interactions were investigated had small sample sizes and lack of genomewide genetic summary measures. Given the fact that a number of genes with small to moderate effects interact with each other in creating susceptibility to psychosis (28), a more comprehensive genetic analysis could provide a more valuable opportunity to analyze these effects. Although this information is currently not available, the DNA samples are preserved for more comprehensive genetic analyses in the future. Furthermore, genetic analyses including the entire cohort would provide more adequate statistical power. Because of limited resources, the nested case-control studies were most optimal. In addition, the broader outcome variable including the subclinical phenotypes and the longitudinal design may help to detect smaller effect sizes. Fourth, although we collected information on history of early childhood adversity, including parental loss, divorce, and separation, more detailed information on history of childhood maltreatment would have provided more comprehensive information on this exposure. False negative findings for childhood adversity thus cannot be excluded. Fifth, general population-based cohort studies represent the naturalistic course of illnesses. It cannot be ruled out that among other variables, treatment modifies the course of an illness. Although we obtained information about the treatment, we cannot rule out that this limitation impacted the results. Finally, as a consequence of the sampling method, both homeless and institutionalized persons could not be included, which may have affected the level of representativeness. However, as both groups are relatively small, effects would be negligible (50).

Analyses of attrition showed interesting results. Unlike what we expected, individuals with a mental health problem at baseline had lower refusal rates at follow-up. Furthermore, there was a difference in the sociodemographic correlates of attrition compared to studies of similar design conducted in western countries because these studies showed higher attrition rates in individuals with a lower socioeconomic status and a lower educational level (11, 76).

## Conclusion

The TurkSch study enabled us to build a comprehensive, high-quality data set on the multidimensional etiological and phenomenological nature of the extended and transdiagnostic psychosis phenotypes. The results of this study, demonstrating associations between baseline variables and noncontact/refusal in the longitudinal arm, are of importance in planning future representative community-based cohort studies in psychiatry. It may be important to make a special effort to include, in future studies, individuals who are younger, more educated, nonmarried, with regular alcohol and *Cannabis* use, and no history of mental health problems.

## Data Availability

The datasets generated for this study are available on request to the corresponding author.

## Ethics Statement

This study was carried out in accordance with the Declaration of Helsinki. All subjects gave written informed consent in accordance with the Declaration of Helsinki. The protocol was approved by the Institutional Ethics Review Board of Ege University.

## Author Contributions

UK, TB, HE, MD, BK, FÖ, HO, KA, and JO conceived and designed the study. UK, TB, HE, BK, and KA collected and collated the data, which were analyzed by UK with supervision from TB, MD, and JO. FO and HO carried out the genetic analysis. All authors were involved in the interpretation of the data. UK drafted the article, which was reviewed and revised by all authors. All authors approved the final version of the manuscript and agreed their accountability in ensuring that any questions related to the accuracy or integrity of any part of the work were appropriately investigated and resolved.

## Funding

This work is a part of the TürkSch project, funded by the Scientific and Technological Council of Turkey 1001 program, project no: 107S053 and 112S476.

## Conflict of Interest Statement

The authors declare that the research was conducted in the absence of any commercial or financial relationships that could be construed as a potential conflict of interest.

## Abbreviations

PE, psychotic experience; PD, psychotic disorder
